# Influence of Surgical Flap Design (Envelope and Szmyd) for Removal of Impacted Mandibular Third Molars on Clinical Periodontal Parameters: A Clinical Trial

**DOI:** 10.3390/ijerph18094465

**Published:** 2021-04-22

**Authors:** Muhtada Ahmad, Zafar Ali Khan, Tahir Ullah Khan, Montaser N. Alqutub, Sameer A. Mokeem, Abdulrahman M. AlMubarak, Mehmood Haider, Mansour Al-Askar, Naseer Ahmed, Nada Aldahiyan, Fahim Vohra, Tariq Abduljabbar

**Affiliations:** 1 Department of Oral & Maxillofacial Surgery, Dow Dental College, Dow University of Health Sciences, Karachi 74200, Pakistan; muhtada.ahmad@duhs.edu.pk; 2 Department of Oral Maxillofacial Surgery and Diagnostic Sciences, College of Dentistry, Jouf University, Sakaka, Al Jouf 72345, Saudi Arabia; drzafar.khan@joden.org; 3 Department of Oral & Maxillofacial Surgery, Lady Reading Hospital Medical Teaching Institute, Peshawar 25000, Pakistan; dr.tahir786@hotmail.com; 4 Department of Periodontics and Community Dentistry, College of Dentistry, King Saud University, Riyadh 11545, Saudi Arabia; alqutub@hotmail.com (M.N.A.); smokeem@ksu.edu.sa (S.A.M.); amalmubarak@ksu.edu.sa (A.M.A.); malaskar@ksu.edu.sa (M.A.-A.); 5 Department of Oral & Maxillofacial Surgery, Abbasi Shaheed Hospital, Karachi 75300, Pakistan; haidermahmoodrv@gmail.com; 6 Department of Prosthodontics, Altamash Institute of Dental Medicine, Karachi 75500, Pakistan; naprosthodontist@gmail.com; 7 Department of Restorative Dental Science, College of Dentistry, King Saud University, Riyadh 11545, Saudi Arabia; naldahiyan@ksu.edu.sa; 8 Department of Prosthetic Dental Science, College of Dentistry, King Saud University, Riyadh 11545, Saudi Arabia; fvohra@ksu.edu.sa

**Keywords:** periodontal attachment loss, periodontal pocket, bone loss, third molar, surgical flaps

## Abstract

The aim of the study was to assess the influence of flap designs (Envelope flap (EF) and Szmyd flap (SF)) for impacted mandibular third molar extraction, on periodontal pocket depth (PPD), clinical attachment loss (CAL) and bone levels (BL) of second molar. Sixty patients indicated for third molar extractions with healthy second molars were allocated into two groups: EF and SF (*n* = 30). Third molars were assessed for angulation, root patterns, depth of impactions and relation with ramus (Pell and Gregory classification). Extraction of third molars was performed and PPD, CAL and BL around second molars at 0, 3 and 6 month (mon) follow-ups (FU) were assessed clinically and radiographically. ANOVA, Chi-square and Fisher’s exact test were employed to compare periodontal factors between EF and SF groups, considering *p* ≤ 0.05 as significant. Sixty participants with a mean age of 23.22 ± 3.17 were included in the study. Based on angulation, the most common impaction in the EF and SF groups was mesio-angular (EF, 50%; SF, 36.7%). Buccal and distal PPD showed a significant increase (*p* < 0.001) in both EF and SF patients from baseline to 6 mon. EF patients showed significantly higher distal and buccal CAL (6.67 ± 0.18 mm; 6.91 ± 0.17 mm) and BL (7.64 ± 0.16 mm; 7.90 ± 0.15 mm) as compared to SF patients (CAL, 6.76 ± 0.26 mm; 6.91 ± 0.17 mm-BL, 7.42 ± 0.38 mm; 7.34 ± 0.34 mm) at 6 mon FU. SF showed better soft tissue attachment (PPD and CAL) and bone stability (less bone loss) around second molars compared to EF after third molar extractions regardless of the patient, tooth and operator factors.

## 1. Introduction

Surgical removal of third molars is a common procedure within the population, with 33% of individuals having at least one removal of the impacted tooth [1]. The treatment planning of extraction is based on the surgical risk, difficulty level, radiographic findings and clinical evidence to avoid possible complications [1,2]. Despite these efforts, clinicians observe several postoperative complications mainly in the mandibular region including, pain, swelling, bleeding, dry socket, trismus, nerve injury and delayed healing, categorized as preventable postoperative complications [3,4]. Thus, to reduce the occurrence of associated postoperative complications clinicians often practice modified extraction techniques.

Knowledge and understanding of surgical concepts are essential and imperative for effective treatment and patient management. Manipulation of the hard and soft tissue during the surgical procedure involves the mucoperiosteal flap reflection, and bone removal for accessibility and ease in extraction [5]. For ease in the extraction, the present literature emphasizes the importance of incision type and flap design for better visibility and accessibility as well as unwanted trauma and healing conditions [6,7,8]. However, the resultant loss of clinical attachment, bone level and increased pocketing are common setbacks associated with the procedure hindering healing over a period [9,10]. Thus, clinicians have established protocols for basic instrumentation, bone removal quantity, flap design and suturing to overcome these limitations.

**Figure 1:**
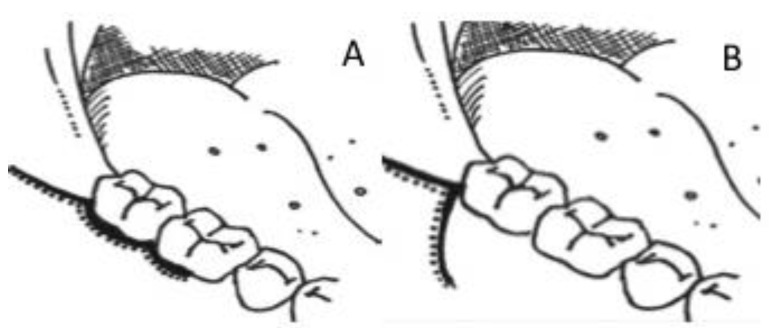
Flap Designs. (**A**) Envelope flap and (**B**) Szmyd flap.

The Envelope and Szmyd flap are the two most commonly advocated flap designs practiced for the removal of the third molar. Each design withholds a unique feature; an envelope with distal relieving incision and the triangular flap with the vestibular extension that is effective for third molar removal at a different angle (Figure 1) [7]. Despite this fact, both techniques result in minimal disruption of the blood supply that aids in the healing process of the reflected flap and wound closure; however, Szmyd flap design provides visibility of the third molar with a decrease in flap tension and stimulates better healing [9]. The Envelope flap imposes a greater risk of periodontal ligament damage and possible pocket formation when creating a sulcular incision around a tooth, increasing osteoclastic activity after flap reflection and wound dehiscence, compared to the Szmyd design [3].

The existing and conflicting literature relevant to preoperative conditions and flap design has influenced the varying outcomes of the periodontal health of the adjacent second molar after third molar impaction surgery. In a study by Rahpeyma et al., it was reported that when comparing the post-operative outcomes of the transposition flap in comparison to the envelop flap in the removal of third molars, the envelop flap showed compromised outcomes [11]. In addition, in a study by Goldsmith et al., the envelop flap was compared to the pedicle flaps in removal of mandibular third molars. Although the pedicle flap showed improvements in some aspects of postoperative outcomes, it also showed increased pain and swelling, and therefore the findings of the study were inconclusive [12]. A debate exists among authors regarding the postoperative conditions that influence the choice of extraction technique for access and healing outcomes [11,12]. Thus, irrespective of the protocol followed, post-operative complications are inevitable for second molars after surgical extraction of third molars. The null hypothesis was that removal of mandibular third molars using Envelope and Szymd flaps will show no difference in bone levels, clinical attachment loss and periodontal pocketing around the second molars. Thus, the present study aims to determine the influence of the two surgical flap designs, namely, the Envelope flap technique and the Szmyd, on the periodontal parameter, including bone level, clinical attachment loss and periodontal pocketing at the second molar after extraction of the mandibular third molar.

## 2. Materials and Methods

### 2.1. Ethical Considerations

The ethics and review board of the Altamash Institute of Dental Medicine provided ethical review and approval for this investigation on 25 October 2019 (Ref No. AIDM/EC/06/2019/10). Participation was voluntary and patients provided written informed consent. The study was performed in accordance with the standards in the Helsinki declaration (2013) and the approved protocol. The right to withdraw from the study was available throughout without any consequences. The trial was reported in accordance with the CONSORT checklist (Appendix A).

### 2.2. Study Design and Participants

The present randomized controlled trial was conducted in the out-patient department of the Medical and Dental College Hospital. A total of 60 patients aged between 18 to 25 years were recruited for the study, and sample size was identified using power calculation (90%), incorporating means and standard deviations from findings of cohorts from previous studies [7,9]. Only patients indicated for lower impacted third molar extraction with the presence of healthy second molars were enrolled in the study; however, patients with concomitant jaw fractures, chronic systemic illness (diabetes mellitus, renal and hepatic disorders, cardiovascular diseases, and patients suffering from the acquired immunodeficiency syndrome or human immunodeficiency virus infection), the habit of smoking, gingivitis and periodontitis, individuals consuming smokeless tobacco products, individuals with maligned dentition, individuals on steroids or non-steroidal anti-inflammatory drugs, probiotics, antibiotics and bisphosphonates within the past 90 days, and preoperative periodontal attachment loss distal to the second molar were excluded from the study.

The selection of patients and treatment planning was based upon the medical, clinical and radiological routine records obtained which included patient’s name, age, gender, presenting complaint, assessment of general health, and clinical and radiological (OPG and periapical radiographs) findings. Patients were randomly allocated (coin toss) (MA &TK), according to the flap design used, into two groups; Envelope (EF) (*n* = 30) and Szmyd (SF) (*n* = 30). Allocation concealment was performed using sealed envelopes and specific individuals (MA and TK) assigned the envelopes (participants) to the study groups.

### 2.3. Questionnaire

A questionnaire was used to collect relevant information on the included participants. Collected information included patient age, gender, oral hygiene and medical history. Information collected in relation to the impacted tooth included presence or absence of caries, angulation of impacted tooth (mesio-angular, disto-angular, horizontal and vertical), root pattern (conical, bulbous, divergent and convergent), depth of impacted molar (Pell and Gregory, Class A, B and C) and tooth relation to the mandible (Pell and Gregory, I, II and III). The duration of the surgical procedure, its details and the operator level were also recorded.

### 2.4. Clinical Assessments

Each patient was clinically assessed in terms of depth of the impacted wisdom tooth, relationship with the ramus of mandible, angulations, root pattern, relationship with the inferior dental canal and periodontal status of the adjacent second molar (both pre- and post-operatively) at 3 and 6 months using a graded probe (Hu-Friedy, Chicago, IL, USA). The periodontal pocket depth (PPD), clinical attachment level (CAL) and alveolar bone loss (BL) along the mid-buccal (buccal) surface and disto-buccal line angle (distal) of the second molar were assessed. The measurement of parameters was carried out to the nearest mm by MA and NA. A digital bitewing radiograph with a standardized long cone parallel method was used to measure the BL in mm. The BL was described as measured from the crest of the bone to the CEJ in a straight line. PPD and CAL measurements were taken under local anesthesia to obtain accurate trans-gingival probing depth. Assessors of clinical parameters (PPD, CAL and BL) were blinded from the clinical procedures performed (MA, NA).

### 2.5. Surgical Procedure

The surgical procedure was conducted under local anesthesia using lignocaine in a 4% solution with 1:100,000 epinephrine. Specialist and consultants in the department of oral surgery performed surgical procedures. For the envelope technique (EF), the incision made extended to the external oblique ridge, up to the midline of the distal line angle of the second molar. Furthermore, a sulcular incision was rendered at the disto-facial line angle of the second molar extending to the first molar mesio-facial line angle. The Szmyd flap technique (SF) followed a similar pattern, however, the length of the incision ran from the disto-facial line angle of the second molar and moved apically towards the mucogingival line, about 2–3 mm. Thus, the technique restricts the involvement of the disto-facial edge of second molar and periodontal tissues when using this technique.

After the incisions, the mucoperiosteal flap was raised and the impacted molar was exposed. The molars were extracted using various surgical instruments including bone drilling as per need. Subsequently, the extraction site was rinsed with saline and sutured back atraumatically, facilitating primary wound healing (Ethicon silk 4-0; Johnson and Johnson, Sao Paulo, Brazil). The interdental suture was placed between second and first molar in addition to 2 or 3 button sutures distal to second molar. Additional sutures were placed in the group for the perpendicular incision. Subsequent to the procedure, all the patients were prescribed 500 mg Amoxicillin (TID) and a follow up after 7 days. Postoperatively, the patients were assessed at 3 and 6 months (mon) follow-up (FU), with clinical and radiographic evaluation, to observe the changes and influence of each technique on periodontal health and bone level.

### 2.6. Statistical Analysis

Data were analyzed using statistical package for social sciences (SPSS version 25, IBM Corporation, Armonk, NY, USA). For the categorical variables, frequencies and percentages were computed, whereas the quantitative variables were calculated and evaluated using means and standard deviations. An independent sample t-test was applied to compare the mean difference between groups based on age. ANOVA, Chi-square test and Fisher’s exact test was employed to compare the difference between the two flap techniques (EF and SF), to compare probing depth, clinical attachment and bone level, considering *p* ≤ 0.05 as significant.

## 3. Results

### 3.1. General Characteristics of Study Participants

Sixty participants (58.3% male and 41.7% female) with a mean age of 23.22 ± 3.17 were included in the study, with 30 subjects in each group (EF and SF) (Table 1 and Table 2). There was no loss of participants in each group. The age range for subjects in EF and SF groups was 21.17 ± 2.21 and 22.27 ± 3.18, respectively; and 51.7% of impacted third molars were on the right side of the mandible, whereas 48.3% of impactions were on the left mandibular side. Based on angulation, the impactions in the EF group were distributed as, mesio-angular 50%, disto-angular 6.7%, horizontal 13.3% and vertical 30%. Similarly, for the SF group, the distribution of impaction angulations was mesio-angular 36.7%, disto-angular 16.7%, horizontal 20% and vertical 26.7%. Based on the depth of impacted molars, the most common class was “A”, comprising 80% and 90% of impactions in EF and SF groups, respectively (Table 3). With regards to the location of impactions to the mandibular ramus, class I was common among the EF (76.7%) and SF (80%) groups. The mean surgical procedure duration for impaction removals among both groups (EF and SF) was reported to be 25.18 ± 6.18 min (Table 2). Seventy percent of impacted teeth in the EF group and 46.7% of impacted teeth among the EF subjects were carious (Table 1). The distribution of subjects and teeth among the EF and SF groups based on gender, site of impactions (right and left), age and caries were statistically comparable (*p* > 0.05) (Table 1). Similarly, a comparable distribution (*p* > 0.05) of impactions among the EF and SF groups on the basis of angulation, root form, depth and ramus relation was observed (Table 3).

### 3.2. Comparison of Periodontal Parameters

**Figure 2:**
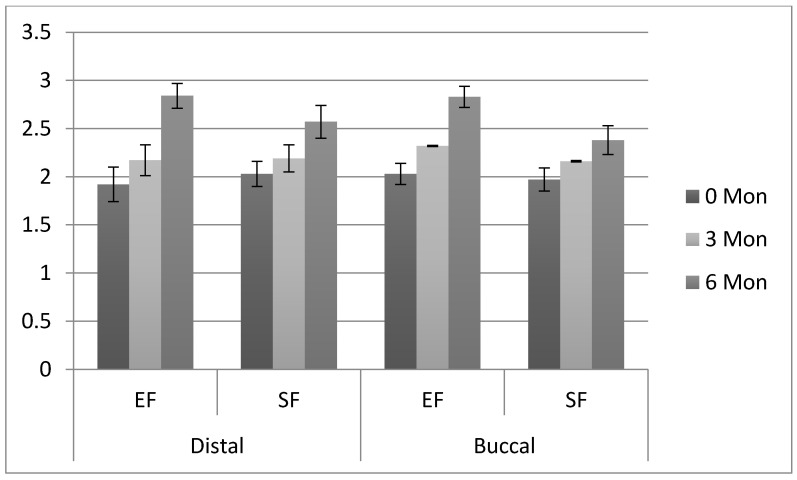
Comparison of means and standard deviations of periodontal pocket depths (mm) observed among the study groups. EF: Envelope flap; SF: Smzyd flap.

The pre-operative distal and buccal second molar (adjacent to the impacted tooth) periodontal pocket depth, clinical attachment level and bone levels among study subjects are presented in Table 4. The increase in distal PPD in the EF group was comparable between baseline (1.92 ± 0.18 mm) and 3 mon (2.17 ± 0.16 mm) FU; however, PPD significantly increased (Table 4) (*p* < 0.005) from 3 mon to 6 mon (2.84 ± 0.13 mm). Similarly, distal PPD among SF patients also showed a significant increase from 3 mon (2.19 ± 0.14 mm) to 6 mon (2.57 ± 0.17 mm) FU (*p* < 0.005). Distal PPD on second molars was significantly higher in EF (2.84 ± 0.13 mm) patients compared to SF patients (2.57 ± 0.17 mm) at 6 mon FU. Buccal PPD showed a significant increase (*p* < 0.001) in both EF and SF patients from baseline (EF (2.03 ± 0.11 mm); SF (1.97 ± 0.12 mm)) to 3 mon (EF (2.32 ± 0.006 mm); SF (2.16 ± 0.008 mm)) and, 3 mon to 6 mon (EF (2.83 ± 0.11 mm); SF (2.38 ± 0.15 mm)) respectively. In addition, buccal PPD was significantly higher (*p* < 0.001) in EF as compared to SF group second molars at 3 mon (EF (2.32 ± 0.006 mm); SF (2.16 ± 0.008 mm)) and 6 mon (EF (2.83 ± 0.11 mm); SF (2.38 ± 0.15 mm)), respectively (Table 4 and Figure 2).

### 3.3. Comparison of Clinical Attachment Loss

**Figure 3:**
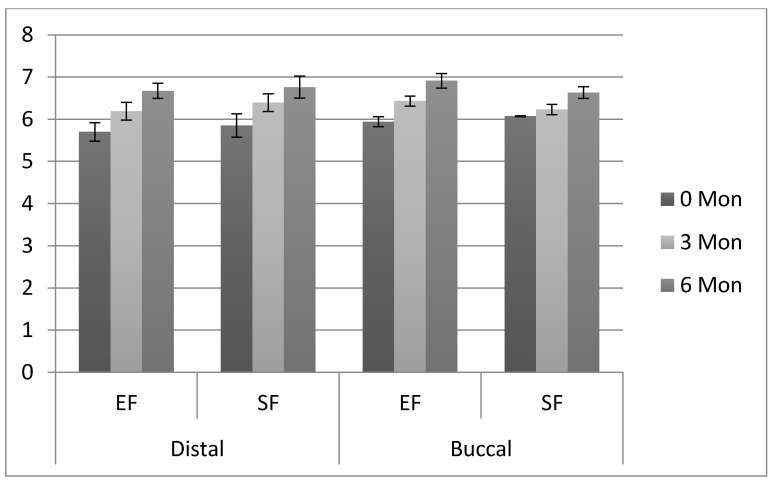
Comparison of clinical attachment loss (means and SD) in mm, among study groups. EF: Envelope flap, SF: Smzyd flap.

Clinical attachment levels for distal and buccal surfaces on the second molars in the EF and SF groups at 3 mon and 6 mon FU are presented in Table 5. The distal CAL in the EF group significantly increased (*p* < 0.05) between baseline (5.70 ± 0.22 mm) and 3 mon (6.19 ± 0.21 mm) and 3 mon to 6 mon (6.67 ± 0.18 mm) FU, respectively (Table 5 and Figure 3). A similar pattern of increase in distal CAL was observed for SF group patients at 3 and 6 mon FU. Distal CAL between EF and SF patients on second molars was statistically comparable at 3 (EF (6.19 ± 0.21 mm); SF (6.39 ± 0.21mm)) and 6 mon (EF (6.67 ± 0.18 mm); SF (6.76 ± 0.26 mm)) FU (*p* > 0.05). The buccal CAL in the EF group significantly increased (*p* < 0.05) between baseline (5.94 ± 0.12 mm) and 3 mon (6.43 ± 0.12 mm) and 3 mon to 6 mon (6.91 ± 0.17 mm) FU, respectively (Table 5). A similar pattern of increased buccal CAL was observed in SF group patients at 3 and 6 mon FU. However buccal CAL was significantly higher (*p* < 0.05) in EF group patients as compared to SF group patients around second molars at 3 (EF (6.43 ± 0.12 mm); SF (6.23 ± 0.12 mm)) and 6 mon (EF (6.91 ± 0.17 mm); SF (6.63 ± 0.14 mm)) FU, respectively.

With regards to distal and buccal bone level (BL), the comparison is presented in Table 6. The distal BL in the EF group significantly increased (*p* < 0.05) between baseline (6.90 ± 0.56 mm) and 3 mon (7.09 ± 0.009 mm) and 3 mon to 6 mon (7.64 ± 0.16 mm) FU, respectively (Table 5). Among the patients in SF group, distal BL around second molars significantly increased (*p* < 0.05) from baseline (6.71 ± 0.32 mm) to 3 mon (7.21 ± 0.29 mm) and 6 mon (7.42 ± 0.38 mm), respectively. Distal BL on second molars was significantly higher in EF patients compared to SF patients at 3 mon (EF (7.09 ± 0.009 mm); SF (7.21 ± 0.29 mm)) and 6 mon (EF (7.64 ± 0.16 mm); SF (7.42 ± 0.38 mm)) FU. The buccal BL in the EF group significantly increased (*p* < 0.05) between baseline (6.79 ± 0.63 mm) and 3 mon (7.39 ± 0.27 mm) and 3 mon to 6 mon (7.90 ± 0.15 mm) FU, respectively (Table 5). A similar pattern of increased buccal BL was seen around second molars in SF patients with increased FU periods (3 and 6 mon). Buccal BL on second molars was significantly higher in EF patients compared to SF patients at 3 mon (EF (7.39 ± 0.27 mm); SF (7.03 ± 0.21mm)) and 6 mon (EF (7.90 ± 0.15 mm); SF (7.34 ± 0.34 mm)) FU (Table 6). The trial was completed in six to seven months.

## 4. Discussion

The present study investigated the influence of the flap design (Envelope flap (EF) and Szmyd flap (SF)) on the periodontal health of adjacent second molars after the extraction of impacted mandibular third molars. The study revealed that, irrespective of the flap design employed, deteriorating periodontal conditions with respect to bone loss, clinical attachment loss and periodontal pocketing were observed. However, a significant difference was observed among the periodontal parameters (PPD, CAL and BL) between the two flap designs with greater BL, PPD and CAL for EF design compared to SF (*p* = 0.001). Thus, the null hypothesis can be partially rejected.

Conventionally, clinicians have employed Envelope flap, as it offers greater field visibility, direct access for third molar extraction and a broad base for vascularity, essential for the healing process [3]. Envelop flap with a distal incision and triangular flap are common approaches for mandibular third molar extractions. Both techniques involve incision over the mucosa for exposure and access, with ostectomy to reach the tooth crown [2,13], and both are widely favored by dental surgeons for mandibular third molar extractions [14]. However, the recent implication of modified flap designs such as Szmyd ensues minimal incisions, limited mucoperiosteum reflection and less postoperative pain, swelling and clinical detachment compared to Envelope design [15,16]. It has been reported that the reflection of the mucoperiosteal flap often has a tendency to strip the mucosa. However, in the present study, no difference was observed in clinical attachment loss between the flap designs distally; however, the difference in flap extension in EF influenced clinical attachment loss buccally. These findings can be related to deficient early regeneration of the connective tissue attachment.

Wood et al. [17] and Rullo et al. [18], reported that bone resorption is an inevitable process upon surgical exposure, irrespective of bone removal. Likewise, iatrogenic damage to the mucosa and bone, particularly for mesiodistal or horizontal angulation extraction, can be considered as a contributing factor for bone resorption. It is suggested that the process of bone removal for accessibility greatly disrupts the hemostasis, resulting in a reduction in the vertical height of the ridge and lingual displacement of the tooth axis irrespective of the technique employed over time [19,20]. According to Nabeeh et al. [8], 43% of bone resorption was observed in EF compared to 19% in SF. Hence, the present study findings correlate with the previous studies indicating the minimal reflection of the SF presented, with lesser bone resorption on the buccal side of the second molar than the distal; hence, reduced pocket depth adjacently was also noted [19,20].

Previous literature provided evidence that the disto-facial aspect of the second molars shows pocketing due to the gaping and wound dehiscence at the incision area [21]. It has been reported that 30% of the wound dehiscence is noted in Envelope flap surgeries compared to 10% wound dehiscence in Szmyd flap design [7]. The present study revealed a significantly greater probing depth in EF compared to SF except on the distal side at 6 mon. The plausible explanation in this regard may be derived from the fact that the inter-sucular suture placed anteriorly is influenced by hematoma pressure, which disturbs the wound margin, increasing odds for wound dehiscence leading to clinical attachment loss and increased probing depth [22,23,24].

Third molar impactions are commonly observed in adults, with ages ranging from 18 to 25 years. Young adults with no periodontal disease have shown better healing capacity regardless of the associated operative factors [24]. It has been reported that patient factors (age and medical status), tooth factors (caries, root pattern, type of impaction and tooth position), and operative factors (e.g., surgical time, the procedure employed and surgeons’ experience) act as confounding elements in exacerbating periodontal conditions [25]. Moreover, authors have established a link between bone removal and nerve injuries with flap design [26,27]. Thus, the depth of the impaction and relation to ramus evaluation has been shown to greatly influence the flap design selection; nevertheless, these differences were not assessed in the present study. However, the present study did not assess significant association of these factors with the worsening periodontal parameters postoperatively.

Within this limitation, the clinical significance of flap design selection for impacted third molar surgery on periodontal health was proven irrespective of the patient, tooth and operative factors. Comparatively to EF, the Szmyd flap presented with better control of the periodontal parameters including bone resorption, clinical attachment loss and periodontal pocket formation. It is pertinent to mention that the assessment period was up to six mon, which does not reflect long-term influence of choice of flap design and quality of healing. In addition, patient cooperation and absence was a limiting factor encountered in the study. Therefore, further studies with long-term follow up of clinical parameters and patient perception of post-surgical outcomes following surgical removal of third molars with a focus on perioperative management are recommended.

## 5. Conclusions

Szmyd flap (SF) design showed better PPD, CAL and BL outcomes for second molars compared to Envelope flap design regardless of the patient, tooth and operative factor. The nominal reflection of the flap in SF design showed better healing conditions under minimal disruption of the blood supply compared to envelop flap.

## Figures and Tables

**Table 1:** Comparison of tooth and subject characteristics in study groups.

Tooth and Subject Characteristics	Envelope	Szmyd	Total	*p*-Value *
Gender				
Male	17 (56.7%)	18 (60%)	35 (58.3%)	0.79
Female	13 (43.3%)	12 (40%)	25 (41.7%)	
Site of impacted molar				
Right	16 (53.3%)	15 (50%)	31 (51.7%)	0.79
Left	14 (46.7%)	15 (50%)	29 (48.3%)	
Caries in 3rd molar				
Yes	21 (70%)	14 (46.7%)	35 (58.3%)	0.067
No	9 (30%)	16 (53.3%)	25 (41.7%)	

* Chi square, *p*-value of >0.05 denote statistically comparable outcomes.

**Table 2:** Characteristics of patients at baseline.

Variables	Envelope Flap (Mean ± SD)	Szmyd Flap (Mean ± SD)	All Patients (Mean ± SD)	Range
Age (Years)	22.50 ± 3.07 ^a^	23.28 ± 3.05 ^a^	23.22 ± 3.17	18–25
Surgical time (minutes)	24.27 ± 5.62 ^a^	25.23 ± 6.22 ^a^	25.18 ± 6.18	36–12
Distal pocket Depth (mm)	1.98 ± 0.18 ^a^	2.03 ± 0.13 ^a^	1.97 ± 0.17	2.40–1.5
Buccal pocket depth (mm)	2.03 ± 0.11 ^a^	1.97 ± 0.12 ^a^	2.00 ± 0.12	2.24–1.5
Distal clinical attachment (mm)	5.70 ± 0.22 ^a^	5.85 ± 0.28 ^a^	5.78 ± 0.26	6.34–5.02
Buccal clinical attachment (mm)	5.94 ± 0.12 ^a^	6.07 ± 0.008 ^a^	6.01 ± 0.12	6.22–5.58
Distal bone level (mm)	6.90 ± 0.56 ^a^	6.71 ± 0.32 ^a^	7.15 ± 0.23	7.90–6
Buccal bone level (mm)	6.79 ± 0.63 ^a^	6.87 ± 0.20 ^a^	6.83 ± 0.47	9.70–6.02

Dissimilar small superscript alphabets in the same row show significant difference. (*p* < 0.05).

**Table 3:** Pre-operative impacted third molar radiographic findings among the study groups.

Radiographic Finding	Envelope (*n* = 30)	Szmyd (*n* = 30)	Total (*n* = 60)	*p*-Value
Angulations				
Mesio-angular	15 (50%)	11 (36.7%)	26 (43.3%)	0.50
Disto-angular	2 (6.7%)	5 (16.7%)	7 (11.7%)	
Horizontal	4 (13.3%)	6 (20%)	10 (16.7%)	
Vertical	9 (30%)	8 (26.7%)	17 (28.3%)	
Root Pattern				
Conical	16 (53.3%)	9 (30%)	25 (41.7%)	0.17
Bulbous	7 (23.3%)	8 (26.7%)	15 (25%)	
Divergent	0 (0%)	2 (6.7%)	02 (3.3%)	
Convergent	7 (23.3%)	11 (36.7%)	18 (30%)	
Depth of impacted molar				
A	24 (80%)	27 (90%)	51 (85%)	0.47
B	6 (20%)	3 (10%)	9 (15%)	
C	0 (0%)	0 (0%)	0 (0%)	
Relation with ramus of mandible				
I	23 (76.7%)	24 (80%)	47 (78.3%)	0.75
II	7 (23.3%)	6 (20%)	13 (21.7%)	
III	0 (0%)	0 (0%)	0 (0%)	

**Table 4:** Comparison of periodontal pocket depth (mm) around second molars included among study groups.

Periodontal Pocket Depth (PPD)	Flap Design	Baseline	3rd Month	6th Month	*p*-Value *
Distal	Envelope	1.92 ± 0.18 ^Aa^	2.17 ± 0.16 ^Aa^	2.84 ± 0.13 ^Ab^	(0 mon–3 mon) 0.094 (0 mon–6 mon) 0.007
	Szmyd	2.03 ± 0.13 ^Ba^	2.19 ± 0.14 ^Aa^	2.57 ± 0.17 ^Bb^	(0 mon–3 mon) 0.073 (0 mon–6 mon) 0.014
*p*-Value ^§^		0.012	0.72	0.0001	
Buccal	Envelope	2.03 ± 0.11 ^Aa^	2.32 ± 0.006 ^Ab^	2.83 ± 0.11 ^Ac^	(0 mon–3 mon) 0.033 (0 mon–6 mon) 0.016
	Szmyd	1.97 ± 0.12 ^Aa^	2.16 ± 0.008 ^Bb^	2.38 ± 0.15 ^Bc^	(0 mon–3 mon) 0.046 (0 mon–6 mon) 0.028
*p*-Value ^§^		0.051	0.001	0.001	

* Post hoc, ^§^ *t* test. Dissimilar superscript capital alphabet in same column (at same location) denotes significant difference (*p* < 0.05). Dissimilar superscript small alphabet in same row (at same location) denote significant difference (*p* < 0.05).

**Table 5:** Comparison of clinical attachment loss (mm) around second molars among study groups.

Clinical Attachment Loss (CAL)	Flap Design	Baseline	3rd Month	6th Month	*p*-Value *
Distal	Envelope	5.70 ± 0.22 ^Aa^	6.19 ± 0.21 ^Ab^	6.67 ± 0.18 ^Ac^	(0 mon–3 mon) 0.036 (0 mon–6 mon) 0.027
	Szmyd	5.85 ± 0.28 ^Aa^	6.39 ± 0.21 ^Ab^	6.76 ± 0.26 ^Ac^	(0 mon–3 mon) 0.020 (0 mon–6 mon) 0.017
*p*-Value ^§^		0.12	0.055	0.08	
Buccal	Envelope	5.94 ± 0.12 ^Aa^	6.43 ± 0.12 ^Ab^	6.91 ± 0.17 ^Ac^	(0 mon–3 mon) 0.034 (0 mon–6 mon) 0.011
	Szmyd	6.07 ± 0.008 ^Ba^	6.23 ± 0.12 ^Bb^	6.63 ± 0.14 ^Bc^	(0 mon–3 mon) 0.446 (0 mon–6 mon) 0.015
*p*-Value ^§^		0.0001	0.0001	0.0001	

* Post hoc, ^§^ *t* test. Dissimilar superscript capital alphabet in same column (at same location) denotes significant difference (*p* < 0.05). Dissimilar superscript small alphabet in same row (at same location) denote significant difference (*p* < 0.05).

**Table 6:** Comparison of bone levels (mm) around second molars among study groups.

Bone Loss	Flap Design	Baseline	3rd Month	6th Month	*p*-Value *
Distal	Envelope	6.90 ± 0.56 ^Aa^	7.09 ± 0.009 ^Ab^	7.64 ± 0.16 ^Ac^	0.01
	Szmyd	6.71 ± 0.32 ^Aa^	7.21 ± 0.29 ^Bb^	7.42 ± 0.38 ^Bc^	0.01
*p*-Value ^§^		0.11	0.03	0.006	
Buccal	Envelope	6.79 ± 0.63 ^Aa^	7.39 ± 0.27 ^Ab^	7.90 ± 0.15 ^Ac^	0.001
	Szmyd	6.87 ± 0.20 ^Aa^	7.03 ± 0.21 ^Bb^	7.34 ± 0.34 ^Bc^	0.001
*p*-Value ^§^		0.512	0.0005	0.0005	

* ANOVA, ^§^ *t* test. Dissimilar superscript capital alphabet in same column (at same location) denotes significant difference (*p* < 0.05). Dissimilar superscript small alphabet in same row (at same location) denote significant difference (*p* < 0.05).

## Data Availability

The data that support the findings of this study are available from the corresponding author, upon reasonable request.

## References

[1] Dolanmaz D., Esen A., Isik K., Candirli C. (2013). Effect of 2 flap designs on postoperative pain and swelling after impacted third molar surgery. Oral Surg. Oral Med. Oral Path. Oral Rad..

[2] Jakse N., Bankaoglu V., Wimmer G., Eskici A., Pertl C. (2002). Primary wound healing after lower third molar surgery: Evaluation of 2 different flap designs. Oral Surg. Oral Med. Oral Path. Oral Rad. Endod..

[3] Erdogan Ö., Tatlı U., Üstün Y., Damlar I. (2011). Influence of two different flap designs on the sequelae of mandibular third molar surgery. Oral Maxfac. Surg..

[4] Monaco G., Daprile G., Tavernese L., Corinaldesi G., Marchetti C. (2009). Mandibular third molar removal in young patients: An evaluation of 2 different flap designs. J. Oral Maxfac. Surg..

[5] Koyuncu B.Ö., Çetingül E. (2013). Short-term clinical outcomes of two different flap techniques in impacted mandibular third molar surgery. Oral Surg. Oral Med. Oral Path. Oral Rad..

[6] Enrico Borgonovo A., Giussani A., Battista Grossi G., Maiorana C. (2014). Evaluation of postoperative discomfort after impacted mandibular third molar surgery using three different types of flap. Quintessence Intern..

[7] Alqahtani N.A., Khaleelahmed S., Desai F. (2017). Evaluation of two flap designs on the mandibular second molar after third molar extractions. J. Oral Maxillofac. Pathol..

[8] Di Nardo D., Mazzucchi G., Lollobrigida M., Passariello C., Guarnieri R., Galli M., De Biase A., Testarelli L. (2019). Immediate or delayed retrieval of the displaced third molar: A review. J. Clin. Exper. Dent..

[9] Yolcu Ü., Acar A. (2015). Comparison of a new flap design with the routinely used triangular flap design in third molar surgery. Int. J. Oral Maxfac. Surg..

[10] Briguglio F., Zenobio E.G., Isola G., Briguglio R., Briguglio E., Farronato D. (2011). Complications in surgical removal of impacted mandibular third molars in relation to flap design: Clinical and statistical evaluations. Quintessence Intern..

[11] Rahpeyma A., Khajehahmadi S., Ilkhani S. (2015). Wound dehiscence after wisdom tooth removal in mandibular mesioangular class IB impactions: Triangular transposition flap versus envelope flap. J. Dent. Res. Dent. Clin. Dent. Prosp..

[12] Goldsmith S.M., De Silva R.K., Tong D.C., Love R.M. (2012). Influence of a pedicle flap design on acute postoperative sequelae after lower third molar removal. Int. J. Oral Maxfac. Surg..

[13] Suarez-Cunqueiro M.M., Gutwald R., Reichman J., Otero-Cepeda X.L., Schmelzeisen R. (2003). Marginal flap versus paramarginal flap in impacted third molar surgery: A prospective study. Oral Surg. Oral Med. Oral Path. Oral Radiol. Endodontol..

[14] Kugelberg C.F. (1990). Periodontal healing two and four years after impacted lower third molar surgery: A comparative retrospective study. Int. J. Oral Maxfac. Surg..

[15] Marciani R.D. (2012). Complications of third molar surgery and their management. Atlas Oral Maxillofac. Surg. Clin. N. Am..

[16] Osunde O., Adebola R., Omeje U. (2011). Management of inflammatory complications in third molar surgery: A review of the literature. Afr. Health Sci..

[17] Wood D.L., Hoag P.M., Donnenfeld O.W., Rosenfeld L.D. (1972). Alveolar crest reduction following full and partial thickness flaps. J. Periodontol..

[18] Rullo R., Addabbo F., Papaccio G., D’Aquino R., Festa V.M. (2013). Piezoelectric device vs. conventional rotative instruments in impacted third molar surgery: Relationships between surgical difficulty and postoperative pain with histological evaluations. J. Cranio-Maxillofac. Surg..

[19] Bello S.A., Adeyemo W.L., Bamgbose B.O., Obi E.V., Adeyinka A.A. (2011). Effect of age, impaction types and operative time on inflammatory tissue reactions following lower third molar surgery. Head Face Med..

[20] Barbosa-Rebellato N.-L., Thomé A.-C., Costa-Maciel C., Oliveira J., Scariot R. (2011). Factors associated with complications of removal of third molars: A transversal study. Med. Oral Patol. Oral Cir. Bucal..

[21] Malkawi Z., Al-Omiri M.K., Khraisat A. (2011). Risk indicators of postoperative complications following surgical extraction of lower third molars. Med Princ. Pract..

[22] Komerik N., Muglali M., Tas B., Selcuk U. (2014). Difficulty of impacted mandibular third molar tooth removal: Predictive ability of senior surgeons and residents. J. Oral Maxillofac. Surg..

[23] Lata J., Tiwari A.K. (2012). Incidence of lingual nerve paraesthesia following mandibular third molar surgery. Natl. J. Maxillofac. Surg..

[24] de Santana-Santos T., de Souza-Santos J.A., Martins-Filho P.R., da Silva L.C., de Oliveira E Silva E.D., Gomes A.C. (2013). Prediction of postoperative facial swelling, pain and trismus following third molar surgery based on preoperative variables. Med. Oral Patol. Oral Cir. Bucal.

[25] Cunha-Cruz J., Rothen M., Spiekerman C., Drangsholt M., McClellan L., Huang G.J. (2014). Recommendations for third molar removal: A practice-based cohort study. Am. J. Public Health.

[26] Sarikov R., Juodzbalys G. (2014). Inferior alveolar nerve injury after mandibular third molar extraction: A literature review. J. Oral Maxillofac. Res..

[27] Nguyen E., Grubor D., Chandu A. (2014). Risk factors for permanent injury of inferior alveolar and lingual nerves during third molar surgery. J. Oral Maxillofac. Surg..

